# Biodegradable Polymers Induce CD54 on THP-1 Cells in Skin Sensitization Test

**DOI:** 10.1155/2011/424571

**Published:** 2011-08-02

**Authors:** Yeon Suk Jung, Reiko Kato, Toshie Tsuchiya

**Affiliations:** ^1^Division of Medical Devices, National Institute of Health Sciences, 1-18-1 Kamiyoga, Setagaya-ku, Tokyo 158-8501, Japan; ^2^Division of Urology, Cancer Institute of New Jersey, Robert Wood Johnson Medical School, New Brunswick, NJ 08901, USA; ^3^Medical Center for Translational Research, Osaka University Hospital, 2-15 Yamadaoka, Suita, Osaka 565-0871, Japan

## Abstract

Currently, nonanimal methods of skin sensitization testing for various chemicals, biodegradable polymers, and biomaterials are being developed in the hope of eliminating the use of animals. The human cell line activation test (h-CLAT) is a skin sensitization assessment that mimics the functions of dendritic cells (DCs). DCs are specialized antigen-presenting cells, and they interact with T cells and B cells to initiate immune responses. Phenotypic changes in DCs, such as the production of CD86 and CD54 and internalization of MHC class II molecules, have become focal points of the skin sensitization test. In this study, we used h-CLAT to assess the effects of biodegradable polymers. The results showed that several biodegradable polymers increased the expression of CD54, and the relative skin sensitizing abilities of biodegradable polymers were PLLG (75 : 25) < PLLC (40 : 60) < PLGA (50 : 50) < PCG (50 : 50). These results may contribute to the creation of new guidelines for the use of biodegradable polymers in scaffolds or allergenic hazards.

## 1. Introduction

Until recently, studies of skin sensitization have used the guinea pig maximization test (GPMT) [1–3]. Currently, the local lymph node assay (LLNA) [4, 5] is often used as an alternative to GPMT. The advantages of LLNA are the ability to make dose-dependent evaluations, a decrease in animal usage, shorter experimental time periods, and lower costs [6, 7]. In addition, a movement to prohibit the use of animals for safety testing of new materials is spreading throughout the world [8]. Recently, Ashikage et al. (2006) [9], Sakaguchi et al. (2006) [10] and several laboratories have reported the use of a new alternative to animal experiments, the human cell line activation test (h-CLAT) [11, 12]. 

Dendritic cells (DCs) are in contact with the skin and inner lining of the nose, lungs, stomach, and intestines [13]. They can also be found in an immature state in the blood. Antigens from viruses and microbes not only induce an adaptive immune response in DCs but also induce innate immunity to activate the immune system [14–16]. When skin sensitization develops, DCs immigrate to the secondary lymphoid organs to present naïve T cells [17]. Then, immature DCs mature and intercellular adhesion molecules, costimulatory molecules, and major histocompatibility complex II (MHC II) antigens (CD54, CD86, HLA-DR antigens) [18–21]. The immune reaction of DCs remains weak without costimulatory molecules. Therefore, skin sensitization in *in vitro* experiments is judged by expressions of CD54 and CD86.

THP-1 cells, a human monocyte cell line, are used in place of DCs in h-CLAT. We can evaluate phenotypical alterations on these THP-1 cells. In the present studies, we assessed skin sensitization to biodegradable polymers by *in vitro* measures.

## 2. Materials and Methods

### 2.1. Cell Cultivation

THP-1 cells purchased from American Type Culture Collection (Manassas, Va, USA) were cultured with RPMI Medium 1640 (GIBCO, Grand Island, NY, USA) supplemented with 10% fetal bovine serum (FBS, Intergen, Purchase, NY, USA), 0.05 mM 2-mercaptoethanol (GIBCO), and 1% streptomycin (GIBCO). The cells used in testing were between 2 weeks to 2 months old.

### 2.2. Biodegradable Polymers and Their Treatment

Biodegradable polymers P1 to P6 (Table 1) were obtained from Taki Chemical Co., Ltd. (Hyogo, Japan). We made 6 initial stock solutions by dissolving the biodegradable polymers in dimethyl sulfoxide (DMSO, Sigma, Ayrshire, UK). The biodegradable polymers P1, P2, P3, P4, P5, and P6 were dissolved separately in DMSO. 2,4-Dinitrochlorobenzene (DNCB, Sigma-Aldrich, St. Louis, Mo, USA) was also dissolved in DMSO and used as a positive control for the skin sensitization test. We made 6 substock solutions of each polymer using two-times dilution series. These sub-stock solutions were diluted 20 times with RPMI medium 1640. Finally, the working solutions were added to the cells at 100 times dilution.

### 2.3. Analysis by Flow Cytometry

Cells were seeded on 96-well plate (Corning, Corning, NY, USA) at a density of 1.6 × 10^5^ (cells/160 *μ*L/well) with the working solutions and cultivated in a CO_2_ incubator for 24 h. The cultivated cells were moved to V-shaped culture plates (BMbio, Tokyo, Japan), and then the cells were collected by centrifuging at 700 ×g for 3 min. The cells were washed 3 times using FACS buffer (PBS+0.1% BSA). They were blocked with 200 *μ*L of 0.01% Globulins Cohn fraction II and III (Sigma-Aldrich, St. Louis, MO, USA) for 15 min on ice. The supernatants were removed from the plate after centrifuging. Fluorescein isothiocyanate (FITC)-conjugated mouse antihuman CD86 antibody (CD86, BD Pharmingen, San Jose, CA, USA), monoclonal mouse anti-human CD54, ICAM-1/FITC antibody, (CD54, Dako, Denmark), or mouse IgG1/FITC antibody (IgG, Dako) was added to the plates, and the plates were shaded from the light for 30 min. The plates were washed 3 times with FACS buffer. Finally, the cells were resuspended in 400 *μ*L FACS buffer and subjected to flow cytometry on a BD FACSCalibur cell sorter (Becton Dickinson Co., Ltd., Franklin Lakes, NJ, USA) to analyze the surface of the cells for CD markers.

### 2.4. Propidium Iodide Assay of Cell Viability

During flow cytometry analysis, propidium iodide (PI, 0.625 *μ*g/mL) was added to the suspended cells to measure the number of living cells. Each test was done in triplicate, and 10,000 living cells were counted each time by the BD FACSCalibur flow cytometer.

### 2.5. Relative Fluorescence Intensity

We separated the living cells from the PI-dyed dead cells using the Flowjo computer program. We evaluated the skin sensitization caused by the biodegradable polymers by measuring the fluorescence intensity of CD86, CD54, and IgG. At less than 50% cell viability, there is a possibility of cell wall damage and irregular binding of antibodies. Therefore, we measured cell viability by calculating the relative fluorescence intensity (RFI) as shown below by the expression levels of CD86 and CD54.

(1) $$\text{RFI}\left(\text{\%}\right)=\frac{\text{MFI}\;\text{of}\;\text{cells}\;\text{with}\;\text{polymer}-\text{MFI}\;\text{of}\;\text{isotype}\;\text{control}\;\text{cells}\;\text{with}\;\text{polymer}}{\text{MFI}\;\text{of}\;\text{vehicle}\;\text{control}\;\text{cells}-\text{MFI}\;\text{of}\;\text{vehicle}\;\text{isotype}\;\text{control}\;\text{cells}}\times 100$$

*MFI: (geometric) mean of fluorescence intensity.

## 3. Results

### 3.1. Cell Viability by PI Assay

**Figure 1:**
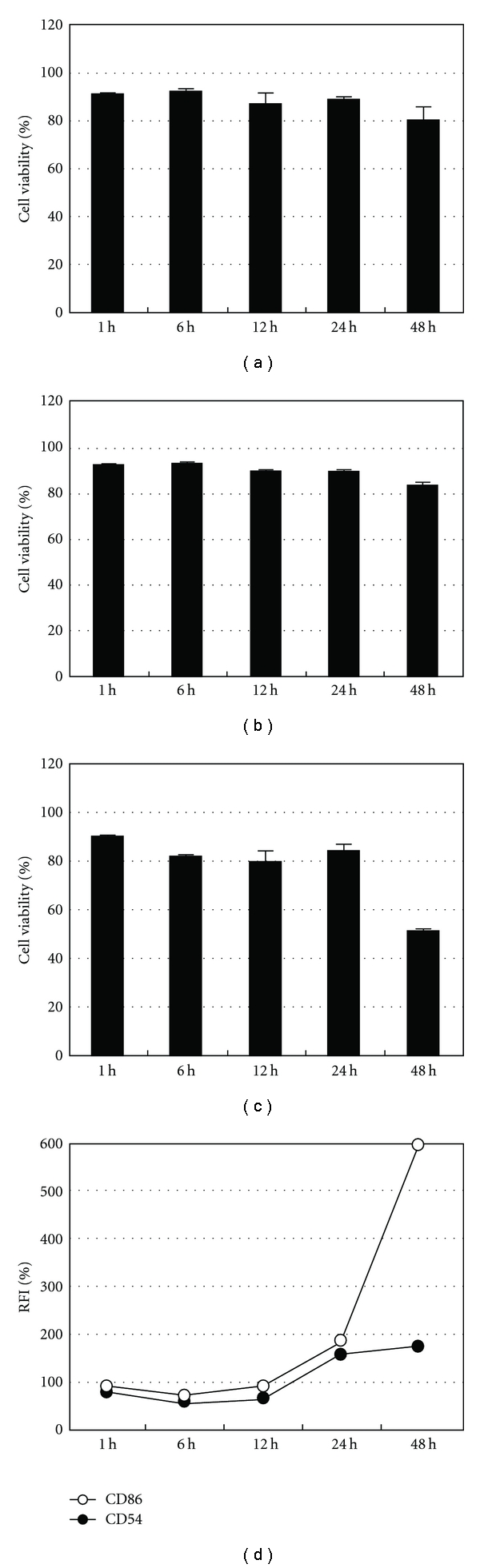
Comparison between cell viabilities of positive control, solvent, and medium only. All cell viabilities were measured by time progression. (a) Cell viability in RPMI medium 1640, (b) cell viability in DMSO (2 *μ*L/mL), (c) cell viability in DNCB (5.2 *μ*g/mL), and (d) CD54 and CD86 expression levels over time with DNCB (5.2 *μ*g/mL).

DNCB, a positive control, was used to test cell viability and expression levels of the CD markers on THP-1 cells. DNCB at 5.2 *μ*g/mL produced high RFI expression levels, while the changes in cell viability were miniscule. At the same time, we tested the effect of the DMSO solvent on cell viability over time (Figure 1). The cell viabilities did not change during the 24-hour incubation with DNCB and DMSO. These results show that a 24-hours incubation and 5.2 *μ*g/mL DNCB were an appropriate incubation time and concentration for the skin sensitization test.

**Figure 2:**
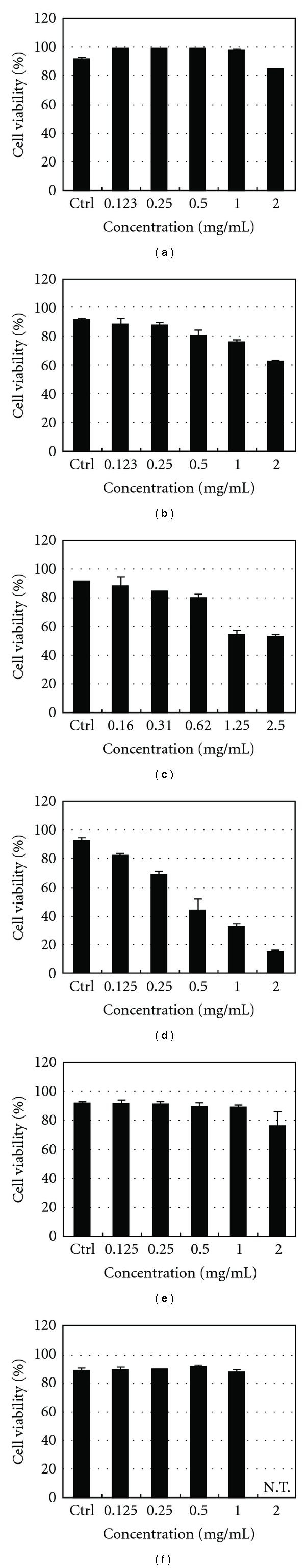
Effects of biodegradable polymers on cell viability by concentration. THP-1 cells were incubated with dissolved biodegradable polymers for 24 hours. Using flow cytometry with PI (0.625 *μ*g/mL), only living cells were counted in total cells. (a) P1, (b) P2, (c) P3, (d) P4, (e) P5, and (f) P6, N.T.: not tested.

Next we tested the effects of biodegradable polymers on the viabilities of THP-1 cells. The cells were incubated for 24 hours with various concentrations of the biodegradable polymers and subjected to PI assay (Figure 2). The results show that cell viabilities differed depending on the biodegradable polymer. However, the tendencies of decreasing cell viabilities were proportional to the concentrations of the polymers. Sample P6 was not tested because it did not dissolve at 2 mg/mL.

### 3.2. Expression of CD54 and CD86 Markers (Alternative RFI)

**Figure 3:**
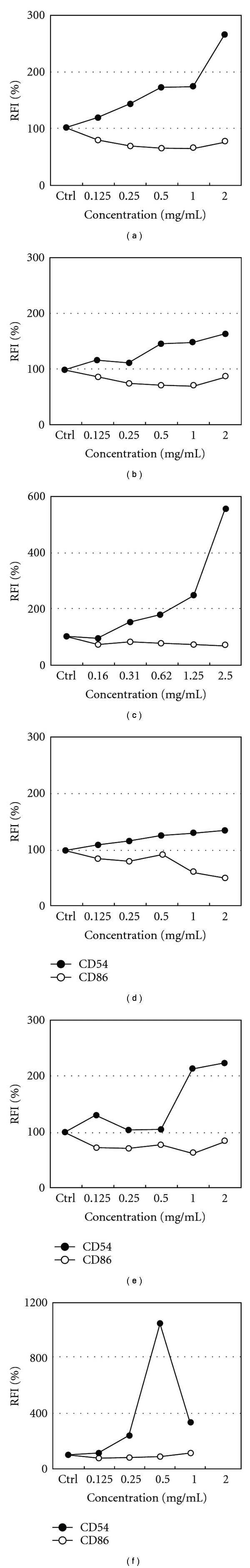
RFI results for each biodegradable polymers. Only living cells were selected by using PI dye. Next, we displayed living cells as FITC histograms, and then we showed the power of the Geom mean. RFI (%) was calculated from Geom mean as described in test. (a) P1, (b) P2, (c) P3, (d) P4, (e) P5, and (f) P6, N.T.: not tested.

After 24-hour incubation, variations of the RFI as an index of skin sensitization were determined by measuring the fluorescence intensity of CD86 and CD54 immune markers. The CD54 RFI increased dose dependently with increased concentration of all polymers (Figure 3). In contrast, the CD86 RFI decreased in all of the polymer solutions. These results showed that as the concentrations of degradable polymers were augmented, only the CD54 marker was expressed. P1, P3, P5, and P6 produced high skin sensitization as shown by the RFI reaching 200. In particular, P6 showed an RFI over 200 at 250 *μ*g/mL.

### 3.3. Estimated Concentration of CD54 in Biodegradable Polymers

From the RFI experiments (Figure 3), we know that some of the biodegradable polymers cause expression of only CD54 costimulatory molecules on the dendritic cells. Based on previous studies, the effective concentrations (EC) 200 (CD54) and EC150 (CD86) became the standard for judging skin sensitization in cases of CD54. P1, P3, P5, and P6 were estimated to cause skin sensitization by this method, at the concentrations of polymers shown in Table 2.

## 4. Discussion

Since the role of DCs in skin sensitization has been known, research using human peripheral blood has expanded, but its use has limits due to the limited number of DCs in the blood [22, 23]. The expression levels of the markers CD54 and CD86 have been shown to be closely related to how sensitive skin is [24, 25]. Previous studies have set RFI value criteria for CD86 (≥150%) and CD54 (≥200%) [26, 27]. In this study, we used h-CLAT as the *in vitro* skin sensitization method and investigated whether biodegradable polymers sensitize skin by using these markers *in vitro*. The expression levels of these markers on cells were first counted by geometric means of flow cytometry, and then the RFI values were calculated by the RFI formula. Polymers that stimulated CD86 at 150% or more and CD54 at 200% or more were judged to be sensitizers as in previous studies.

Initially, we tested the effects of DNCB and DMSO on cell viability over time. When the cell viability of the medium concentration group was assumed to be 100%, the cell viability of the DMSO group showed only minuscule change. After 48 hours, the cell viability of both the DMSO group and the medium concentration group had decreased only slightly. The results showed that the cell viability was not changed by the solvent DMSO (Figure 1). Further, the addition of 5.2 *μ*g/mL of DNCB to the cells did not result in a large change in cell viability up to 24 hours. At 48 hours, however, the cell viability had decreased to less than 60%. Therefore, using DNCB as the positive control for skin sensitization was most appropriate when the marker was measured after 24 hours.

We cultured all samples with biodegradable polymers and measured the cell viability and expression levels of CD54 and CD86 at 24 hours. Each polymer exerted a different effect on cell viability (Figure 2). We found that polymer P4 sharply decreased the cell viability as the dosage was increased; therefore, it was possibly the most toxic biodegradable polymer. The polymers P1 and P5 at the highest concentration showed cell viabilities of 70% or more. Therefore, their effect on cell viability is considered weak.

The polymers increased the expression of the CD54 marker but did not increase the expression of the CD86 marker (Figure 3). The P1, P3, P5, and P6 polymers stimulated high expression levels of the CD54 marker. P1 and P5 stimulated high expression levels of the CD54 marker while not affecting cell viability. P4 produced the lowest expression of the CD54 marker and the lowest cell viability of all the polymers. These results suggest that the biodegradable polymers affect only the CD54 marker and differ in their composition.

During the past several decades, biodegradable polymers have been used in clinical medicine, and they have been reported to have minimal to more substantial side effects [28, 29]. Generally, it is known that there are delayed antigen-antibody reactions due to partial wear-out and inflammation due to long usage of a biomedical material. This research may suggest that there is a relationship between CD54 and delayed antigen-antibody reactions. It is thought to be necessary to compare the results of the effects of biodegradable polymers on dendritic cells with those of local lymph node assays (LLNA).

## 5. Conclusion

Until now, biodegradable polymers have been little studied as causes of *in vitro* sensitization. We tested biodegradable polymers by h-CLAT. We could not assess P4 because even a low concentration of P4 caused cell death. Further, skin sensitization was not increased by P2. Biodegradable polymers that showed evidence of causing skin sensitization expressed as only CD54 were PLLG (75 : 25) < PLLC (40 : 60) < PLGA (50 : 50) < PCG (50 : 50). We determined that the different compositions of biodegradable polymers produced clearly different effects on skin sensitization. In conclusion, skin sensitization due to exposure to the biodegradable polymers can be examined without animal experiments.

## Figures and Tables

**Table 1:** Condition and composition of biodegradable polymers.

Sample	Biodegradable polymer	Composition	Mn*	Catalyst
P1	Poly(_L_-lactide-glycolide copolymer)(PLLG)	75 : 25	3540	Without
P2	Poly(_L_-lactide-glycolide copolymer)(PLLG)	75 : 25	3580	SnOct_2_
P3	Poly(_X,L_-lactide-glycolide copolymer)(PLGA)	50 : 50	3550	Without
P4	Poly(_L_-lactide)(PLLA)	100	3390	Without
P5	Poly(_L_-lactide-caprolactone copolymer)(PLLC)	40 : 60	3110	Without
P6	Poly(caprolactone glycolide copolymer)(PCG)	50 : 50	3000	Without

*number average molecular weight (Mn).

**Table 2:** Effects of EC 200 on biodegradable polymers.

Sample	Low FRI	Low conc.	High RFI	High conc.	EC (mg/mL)
	a	b	c	d	
P1	174.00	1.00	265.00	2.00	1.29
P3	178.00	0.62	250.00	1.25	0.81
P5	104.00	0.50	212.00	1.00	0.94
P6	108.00	0.13	236.00	0.25	0.21

Formula: EC200 = (d − b)/(c − a) × (200 − a) + b.
